# The Public School Boy's Body

**Published:** 1892-10-22

**Authors:** 


### Editor's Letter-Box.

[Our correspondents are reminded that prolixity is a great bar to publication, and that brevity of style and conciseness of statement greatly facilitate early insertion.]

### THE PUBLIC SCHOOL BOY'S BODY.

Sir,—Your issue of October 1st has a most valuable article on which I wish to make a few comments on public school dietary. I hope you will not rest until you have thoroughly aroused attention: more than that you may not achieve. Let me go back over twenty years, and give my own experience. The following confirms your article. So far as I know, there is not a single official resident on the spot, nor do I think my remarks can be considered an attack upon any one. I was in a training college, where the age of entrance averaged twenty years, and the tuition two years each person. For growing young men condemned to be "lectured" six hours per day, four hours per diem for private study and preparation of lectures, our diet was: Breakfast, 8 a.m., weak tea or coffee, a portion of butter no larger than an ordinary chestnut or Brazil nut, with unlimited quantity of bread. For tea at 6 30, we had the same allowance—our sugar and milk being a fixed quantity. Dinner at 1.15, we had generally an unlimited supply of the common parts of beef and mutton (rough contract kinds), the second courses of beef, &c., being generally cold, and a very sparse quantity of vegetables (potatos being very dear), and green vegetables; cabbages as usual were served out very sparingly—in my opinion deficient in quantity to keep young men healthy, together with puddings of a varied kind. Supper we had none. Now, sir, I think you do well to draw attention to the need of reform in diet. Had the cost of the meat wasted been applied in variety of supplementary foods, I believe comfort and health would have been better provided for than they were. Good must arise from your article.—Yours truly,

Ex-Schoolmaster.

